# P-2314. Assessing Hazard Ratio of Recurrent Spontaneous Bacterial Peritonitis: Impact of Antibiotic Prophylaxis in Cirrhotic Patients

**DOI:** 10.1093/ofid/ofae631.2466

**Published:** 2025-01-29

**Authors:** Trong-Tuong Nguyen, Niki Arab, Brian Kim, Arthur Jeng

**Affiliations:** Olive View-UCLA Medical Center, Sylmar, California; Olive View- UCLA Medical Center, Los Angeles, California; Olive View-UCLA Medical Center, Sylmar, California; Olive View UCLA Medical Center/UCLA School of Medicine, Sylmar, California

## Abstract

**Background:**

Spontaneous bacterial peritonitis (SBP) is a severe infection of the ascitic fluid in individuals with advanced liver disease and ascites. The American Association for the Study of Liver Diseases recommends antibiotic (abx) prophylaxis (ppx) for patients with a history of SBP, low ascitic fluid protein (< 1.5 g/dL), or advanced cirrhosis with ascites. However, prophylactic antibiotics cause collateral damage and their efficacy in reducing SBP recurrence remains uncertain. This study assesses the recurrence rates between groups receiving prophylaxis and those without.

**Methods:**

Single centered, retrospective cohort study aimed to estimate the hazard ratio (HR) for recurrent SBP in patients with and without SBP ppx. Patients meeting criteria of cirrhosis with documented ascites and polymorphonuclear leukocyte count ≥ 250 cells/µL were randomly selected between 2017 to 2023. The primary outcome was time to recurrent SBP. Cox proportional hazard models were used to estimate the HR and 95% confidence intervals (CIs).

**Results:**

38 patients were included, 4 patients received SBP ppx. The HR for recurrent SBP was not statistically significant [0.765 (95% CI -1.58, 1.04)] between use of SBP ppx and those with no ppx abx. This analysis was controlled for age, gender, alcohol use, cancer, diabetes, chronic kidney disease, chronic lung disease, autoimmune disease, and HIV status. Incidentally we found that, of the 4 patients who received prophylaxis, 2 developed extended spectrum beta lactamase (ESBL) producing gram negative infections in either ascitic or blood cultures.

**Conclusion:**

Our analysis did not demonstrate a statistically significant difference in the hazard of recurrent SBP between patients with versus without ppx. SBP ppx was found to have potential risk of recurrent SBP with more resistant ESBL-producing pathogens for those on SBP ppx. Additional study may be warranted to further elaborate the benefit vs risk of SBP ppx in terms of development of resistance.

**Disclosures:**

All Authors: No reported disclosures

